# Mitral annular plane systolic excursion by cardiac MR is an easy tool for optimized prognosis assessment in ST-elevation myocardial infarction

**DOI:** 10.1007/s00330-019-06393-4

**Published:** 2019-08-07

**Authors:** Agnes Mayr, Mathias Pamminger, Martin Reindl, Simon Greulich, Sebastian J. Reinstadler, Christina Tiller, Magdalena Holzknecht, Timo Nalbach, David Plappert, Christof Kranewitter, Gert Klug, Bernhard Metzler

**Affiliations:** 1 University Clinic of Radiology, Medical University Innsbruck, Anichstraße 35, A-6020 Innsbruck, Austria; 2 University Clinic of Internal Medicine III, Cardiology and Angiology, Medical University Innsbruck, Anichstraße 35, A-6020 Innsbruck, Austria; 3 Department of Cardiology and Cardiovascular Diseases, University Hospital Tübingen, Otfried Müller-Straße 10, 72076 Tübingen, Germany

**Keywords:** Magnetic resonance imaging, ST-elevation myocardial infarction, Prognosis

## Abstract

**Objectives:**

The purpose of this study was to assess the comparative prognostic value of mitral annular plane systolic excursion (MAPSE) versus left ventricular ejection fraction (LVEF), measured by cardiac magnetic resonance (CMR) imaging in patients with ST-elevation myocardial infarction (STEMI) treated with primary percutaneous coronary intervention (pPCI).

**Methods:**

CMR was performed in 255 STEMI patients within 2 days (interquartile range (IQR) 2–4 days) after infarction. CMR included MAPSE measurement on CINE 4-chamber view. Patients were followed for major adverse cardiovascular events (MACE)—death, non-fatal myocardial re-infarction, stroke, and new congestive heart failure.

**Results:**

Patients with MACE (*n* = 35, 14%, median follow-up 3 years [IQR 1–4 years]) showed significantly lower MAPSE (8 mm [7–8.8] vs. 9.6 mm [8.1–11.5], *p* < 0.001). The association between decreased MAPSE (< 9 mm, optimal cut-off value by c-statistics) remained significant after adjustment for independent clinical and CMR predictors of MACE. The AUC of MAPSE for the prediction of MACE was 0.74 (CI 95% 0.65–0.82), significantly higher than that of LVEF (0.61 [CI 95% 0.50–0.71]; *p* < 0.001).

**Conclusions:**

Reduced long-axis function assessed with MAPSE measurement using CINE CMR independently predicts long-term prognosis following STEMI. Moreover, MAPSE provided significantly higher prognostic implication in comparison with conventional LVEF measurement.

**Key Points:**

• *MAPSE determined by CMR independently predicts long-term prognosis following STEMI.*

• *MACE-free survival is significantly higher in patients with MAPSE ≥ 9 mm than < 9 mm.*

• *MAPSE provides significantly higher prognostic implication than conventional LVEF.*

## Introduction

Acute myocardial ischemia has a profound impact on myocardial structure and function [1, 2]. Left ventricular ejection fraction (LVEF) serves as a well-established and robust predictor for worse clinical outcome in patients suffering from acute ST-elevation myocardial infarction (STEMI) [3]. However, propulsion of the intraventricular blood pool is based on a complex interplay of longitudinal shortening, circumferential contraction, and torsion along the long axis of the left ventricle [4]. The main factor for sufficient LV function is ventricular longitudinal shortening, accounting for about 60% of LV stroke volume both in healthy subjects and in disease, including STEMI [5–7]. Therefore, atrioventricular plane motion has been suggested as an easy-to-measure surrogate for ventricular function and it has been shown that reduced amplitude of valvular plane movement in echocardiography is a predictor of adverse events in patients with various cardiovascular diseases [8]. Impaired mitral annular plane systolic excursion (MAPSE) in cardiac magnetic resonance (CMR) imaging is an independent determinant of all-cause mortality in patients with reduced LVEF (< 50%), regardless of the underlying cause of impaired systolic function [9]. Rangarajan et al highlighted a decreased MAPSE as determined by CMR to predict major adverse cardiac events (MACE) in a mixed population, including patients with known coronary artery disease or prior myocardial infarction [10]. However, the prognostic implications of CMR-derived MAPSE in a large homogenously treated patient cohort with acute STEMI have not been assessed so far. The aim of this study was therefore to investigate whether CMR-determined MAPSE predicts MACE in patients with reperfused first-time STEMI and to evaluate its prognostic value in comparison with routine LVEF measurement.

## Methods

### Study population

In this prospective observational study, 289 STEMI patients presenting at the coronary care unit of the Innsbruck University Hospital (Innsbruck, Austria) were initially included. After exclusion of 34 patients, a final cohort of 255 STEMI patients was analyzed. Reasons for exclusion were missing LVEF measurements due to limited image quality caused by arrhythmia and breathing artifacts (*n* = 10) or incomplete short-axis cine coverage of the LV image stack (*n* = 3), inadequate imaging plane for septal MAPSE measurements due to inclusion of the LV outflow tract or aortic valve with subsequent invisibility of the septal insertion point of the mitral valve (*n* = 9), lack of follow-up accessibility because of a changed telephone number or withdrawal of consent to further data acquisition (*n* = 11) or refusion of contrast agent application (*n* = 1).

The following inclusion criteria were applied: first STEMI according to the redefined European Society of Cardiology/American College of Cardiology committee criteria [11] revascularization by primary percutaneous coronary intervention (pPCI) within 24 h after the onset of symptoms, an estimated glomerular filtration rate > 30 mL/min per 1.73 m^2^, and Killip class < 3 at time of CMR. Exclusion criteria were age < 18 years, any history of previous myocardial infarction or coronary intervention, and any contraindication to CMR examination (pacemaker, claustrophobia, orbital foreign body, cerebral aneurysm clip, or known or suggested contrast agent allergy to gadolinium).

The clinical endpoint of the present study was the occurrence of MACE defined as a composite of all-cause death, non-fatal myocardial re-infarction, stroke, and congestive heart failure. Re-infarction was defined in accord with the redefined European Society of Cardiology/American College of Cardiology committee [11] and new congestive heart failure was defined as the first episode of cardiac decompensation requiring diuretic therapy. Follow-up data for clinical endpoint assessment were collected at 6 months and 12 months after STEMI then annually via telephone interview using a standardized questionnaire [12]. All interviews were performed by trained personnel blinded to baseline CMR, laboratory, and angiographic findings. The declared endpoints were checked afterwards by carefully reviewing the corresponding medical records.

Before inclusion in the present study, all participants gave their written informed consent. The study was approved by the local research ethics committee and conducted in conformity with the Declaration of Helsinki.

### Cardiac magnetic resonance imaging

All patients were investigated on a clinical 1.5-T MR imaging unit (AVANTO_fit; Siemens Healthineers) within 2 days (interquartile range (IQR) 2–4 days) after successfully reperfused first acute STEMI by pPCI. The standardized imaging protocol of our research group was published in detail previously [13]*.* Briefly, LV volumes and function were assessed on short-axis cine images using breath-hold, retrospective ECG-triggered trueFISP bright-blood sequences. For postprocessing, standard software (ARGUS; Siemens) was applied. Papillary muscles were assigned to the LV volume [14].

**Fig. 1:**
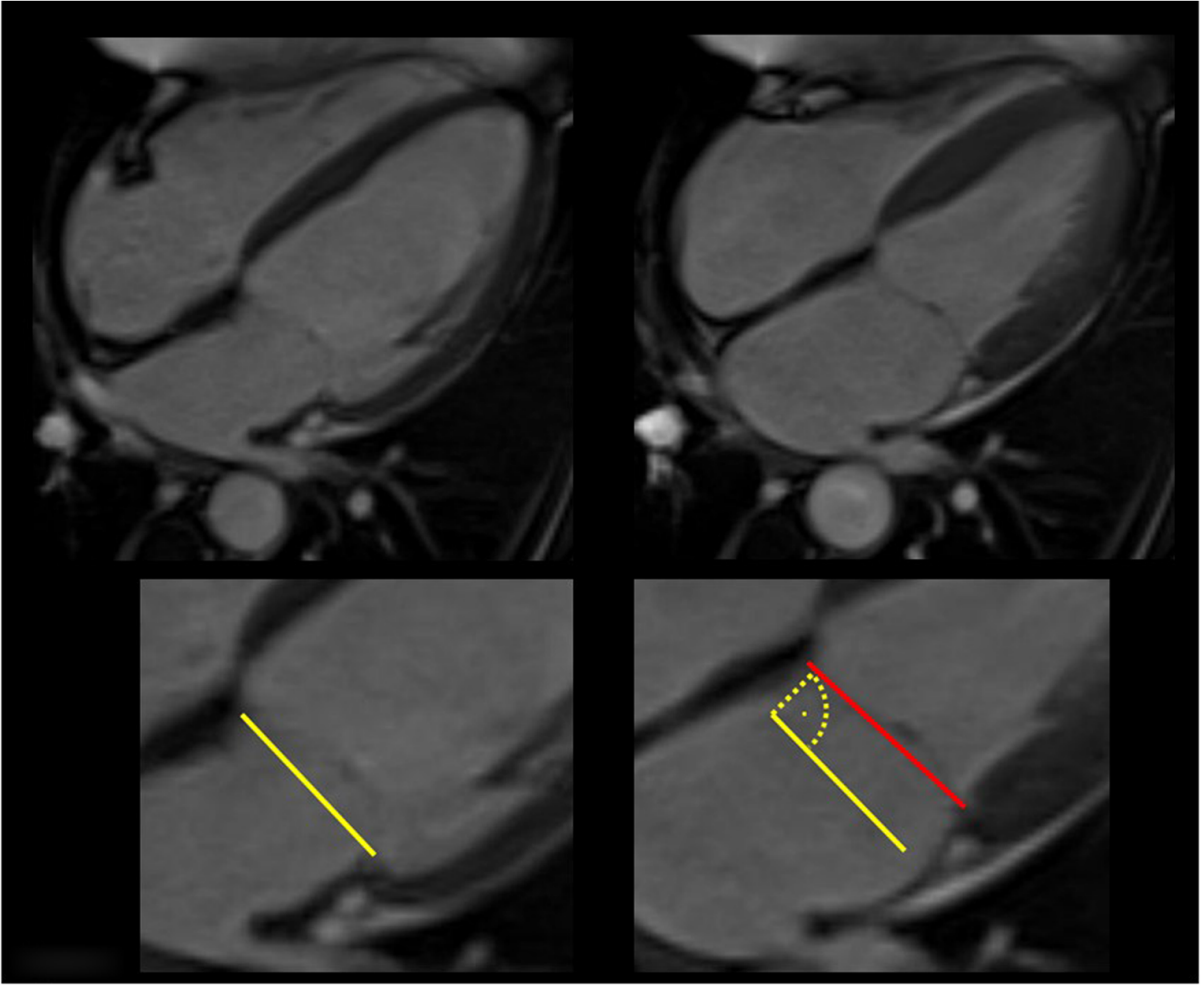
End-diastolic (solid yellow line) and end-systolic mitral annular plane (red line) were defined on a long-axis four-chamber view by connecting the septal and lateral attachment of the mitral valve to the myocardium on the respective images of the long-axis stack, with end-diastole being defined by the largest diameter of the left ventricle and end-systole as the image immediately before mitral valve opening. MAPSE (dotted yellow line) was defined as the perpendicular distance of the end-systolic mitral annular plane to the end-diastolic plane, measured in regard to the septal attachment of the mitral valve in end-diastole

End-diastolic and end-systolic mitral annular planes were defined on a long-axis four-chamber view by connecting the septal and lateral attachment of the mitral valve to the myocardium on the respective images of the long-axis stack, with end-diastole being defined by the largest diameter of the left ventricle and end-systole as the image immediately before mitral valve opening. “Septal” MAPSE was defined as the perpendicular distance of the end-systolic mitral annular plane to the end-diastolic plane, measured in regard to the septal attachment of the mitral valve in end-diastole as shown in Fig. 1. Similarly, “lateral” MAPSE was defined as the distance of the lateral attachment of the mitral valve between end-diastole and end-systole. Average MAPSE is the calculated mean between septal and lateral MAPSE.

Late gadolinium enhancement images were acquired 15 min after the application of a 0.2 mmol/kg bolus of contrast agent (Multihance; Bracco) using an ECG-triggered phase-sensitive inversion recovery sequence with consecutive short-axis slices. The late gadolinium enhancement extent of each slice was quantified by using a PACS workstation (IMPAX; Agfa HealthCare). We defined “hyperenhancement” as + 5 SDs above the signal intensity of remote myocardium in the opposite myocardial segment of the LV [15]. Infarct size (IS) was expressed as a percentage of LV myocardial mass. Microvascular obstruction (MVO) was defined as a persisting area of “hypoenhancement” within the infarcted, hyperenhanced territory [16]. All CMR images were analyzed by experienced observers, blinded to clinical and angiographic data. A randomly determined sample of 30 study participants was evaluated three times to evaluate intra-observer and inter-observer variability.

### Statistical analysis

SPSS Statistics (version 24.0; IBM Corp) and MedCalc (Version 15.8; MedCalc Software bvba) were used for statistical analyses. According to the presence or absence of normal distribution, continuous variables are presented as mean ± SD or median with corresponding IQR. Categorical variables are expressed as absolute numbers and percentages. The differences in continuous variables between 2 groups were evaluated by the Mann–Whitney U test or Student *t* test, as appropriate. The chi-square test was used to assess the differences in categorical variables. Spearman test was applied to calculate correlations of continuous variables. All parameters included in Table 1 were included in univariable Cox regression analyses. Variables with a *p* < 0.10 in univariable analysis and age were entered in a multivariable model [12]. Two different multivariate models were compiled to ensure statistical robustness with respect to our sample size and event rate. In addition to the multivariable model comprising continuous variables (raw data), we formed a dichotomized model including the variables dichotomized according to the optimal cut-off determined by C-statistics to warrant an accurate comparison of hazard ratios. Together with MAPSE, age, the presence of hypertension, and infarct localization were entered in a model of clinical risk factors. Likewise, the presence of MVO and LVEF together with MAPSE were included in a model of imaging risk factors. Area under the curve (AUC) differences were appraised by a method established by de Long et al [17]; accordantly, the AUC values were interpreted using the following standard categories: negligible (≤ 0.55), small (0.56–0.63), moderate (0.64–0.70), and strong (≥ 0.71).

**Table 1:** Patients characteristics

	Total population (*n* = 255)	MACE (*n* = 35, 13.7%)	No MACE (*n* = 220, 86.3%)	*p* value
Age, years	57 (±11)	61.7(±11)	56 (±11)	*0.01*
Female, *n* (%)	40 (15.7)	6 (17.1)	34 (15.5)	0.80
Body mass index (kg/m^2^)	26.1 [24.4–28.3]	26 [24–28]	26.1 [24.4–28.4]	0.76
Diabetes, *n* (%)	21 (8.2)	5 (14.3)	16 (7.3)	0.18
Hyperlipidemia, *n* (%)	156 (61.2)	25 (71.4)	131 (59.5)	0.20
Smoking, *n* (%)	136 (53.3)	15 (42.9)	121 (55)	0.27
Hypertension, *n* (%)	139 (54.5)	25 (71.4)	114 (51.8)	*0.04*
Positive family history, *n* (%)	73 (28.6)	7 (20)	66 (30)	0.31
Peak hs-cTnT (ng/L)	3594 [275–6884]	4257 [23.5–10166]	3471 [298.3–6672]	0.27
LVEF (%)	54.4 [48–59.6]	51.1 [42.2–57.3]	54.5 [48.8–59.7]	*0.05*
LVEDV (mL)	147.9 [120.9–167.6]	153.5 [115.8–166.9]	147.1 [121.4–168.9]	0.81
LVESV (mL)	67.8 [50–82.5]	70.5 [52.9–98.2]	67.2 [50–81.7]	0.28
LV mass (g)	136.7 [115.5–157.6]	143.3 [122.5–160.2]	136.5 [115.4–156.3]	0.38
Septal MAPSE (mm)	9.4 [7.9–11.3]	8 [7–8.8]	9.6 [8.1–11.5]	*< 0.001*
Lateral MAPSE (mm)	11.1 [9.2–13.1]	9.9 [7.6–11.5]	11.4 [9.4–13.2]	*0.002*
Average MAPSE (mm)	10.2 [8.6–12]	8.9 [7.4–10.1]	10.5 [8.9–12.2]	*< 0.001*
IS, % of LVMM	16.9 [7.3–23.8]	15.9 [10.6–21.8]	13.5 [6.9–24]	0.32
MVO, *n* (%)	131 (51.4)	23 (65.7)	108 (49.1)	*0.07*
Infarct localization				*0.04*
Anterior (LAD)	118 (46.3)	22 (62.9)	96 (43.6)	
Non-anterior (RCA and LCX)	137 (53.7)	13 (34.1)	124 (56.4)	

hs-cTnT, high-sensitivity cardiac troponin T; LVEF, left ventricular ejection fraction; LVEDV, left ventricular end-diastolic volume; LVESV, left ventricular end-systolic volume; MAPSE, mitral annular plane systolic excursion; IS % of LVMM, infarct size in percent of left ventricular myocardial mass; MVO, microvascular obstruction; LAD, left anterior descending artery; RCA, right coronary artery; LCX, left circumflex artery

MACE-free survival was estimated and depicted by the Kaplan–Meier method, and differences were assessed by the log-rank test.

Intra-observer and inter-observer variabilities of MAPSE measurements were determined by intraclass correlation coefficients (ICC) and coefficients of variation (CoV). A *p* value of < 0.05 was defined as statistically significant.

## Results

### Subject characteristics

We included 255 consecutive STEMI patients with a total ischemia time of 205 (IQR, 132–351) minutes. Mean age of the overall population was 57 (± 11) years. Baseline characteristics and CMR parameters of the overall cohort are listed in Table 1.

The median of septal MAPSE was 9.4 mm (IQR 7.9–11.3 mm) and median LVEF was 54.4% (IQR 48–59.6%). Acute IS in % of LV myocardial mass was 16.9% (IQR 7.3–23.8%) and MVO was detected in 131 patients (51.4%).

### Determinants of MACE

Thirty-five (13.7%) patients experienced a MACE event (8 deaths, 14 myocardial re-infarctions, 8 strokes, 5 congestive heart failures). Median follow-up time was 3 years (IQR 1–4 years). The median time to event was 36 months (IQR 12–53 months). Table 1 provides all parameters separately for patients with (*n* = 35; 14%) and without (*n* = 220; 86.3%) MACE. There was a significant association of age (*p* = 0.01) and the incidence of hypertension (*p* = 0.04) as well as infarct localization (*p* = 0.04) with the occurrence of MACE. Regarding CMR parameters, patients displaying MACE showed lower LVEF (*p* = 0.05) and MAPSE (*p* < 0.001) and displayed a trend to a higher incidence of MVO (*p* = 0.07). No significant association of MACE with other CMR parameters (IS, LV end-diastolic volume, and LV end-systolic volume) or clinical parameters (diabetes, hyperlipidemia, smoking, positive family history, peak high-sensitivity cardiac troponin T, and infarct-related artery) were found (all *p* > 0.05). In multivariable analysis, MAPSE remained a significant independent predictor of MACE, in both clinical risk factor model (hazard ratio = 0.77 [CI 95% 0.66–0.90]; *p* = 0.001) and CMR risk factor model (hazard ratio = 0.83; CI 95% 0.73–0.95, *p* < 0.006), see Table 2.

**Table 2:** Cox regression analysis for the prediction of MACE

	Univariable analysis		Multivariable analysis	
	Hazard ratio (95% CI)	*p* value	Hazard ratio (95% CI)	*p* value
Clinical risk factor model				
Age	1.021 (0.989–1.054)	*0.19*	–	–
Hypertension	1.410 (0.660–3.014)	0.38	–	–
Infarct localization	2.283 (1.137–4.585)	*0.02*	–	–
MAPSE	0.803 (0.706–0.914)	*< 0.001*	0.770 (0.658–0.901)	*0.001*
CMR risk factor model				
MVO	1.880 (0.935–3.782)	0.07	–	–
LVEF	0.950 (0.917–0.985)	*0.05*	–	–
MAPSE	0.796 (0.710–0.893)	*< 0.001*	0.829 (0.726–0.947)	*0.006*

CI, confidence interval; MAPSE, mitral annular plane systolic excursion; CMR, cardiac magnetic resonance; MVO, microvascular obstruction; LVEF, left ventricular ejection fraction

### Prognostic value of MAPSE

After adjustment for both clinical and imaging risk factors that were univariate predictors (*p* < 0.10), MAPSE < 9 mm remained a significant predictor of MACE in the model of dichotomized clinical risk factors (hazard ratio = 6.02; CI 95% 2.47–14.69, *p* < 0.001) as well as in the model of dichotomized imaging risk factors (hazard ratio = 5.03; CI 95% 2.11–12.01, *p* < 0.001), see Table 3.

**Table 3:** Cox regression analysis for the prediction of MACE (dichotomized clinical and CMR risk factors)

	Univariable analysis		Multivariable analysis	
	Hazard ratio (95% CI)	*p* value	Hazard ratio (95% CI)	*p* value
Clinical risk factor model				
Age > 60	1.338 (0.674–2.659)	0.41	–	–
Hypertension	1.537 (0.719–3.287)	0.27	–	–
Infarct localization	1.805 (0.899–3.622)	0.10	–	–
MAPSE < 9 mm	5.056 (2.182–11.716)	*< 0.001*	6.021 (2.469–14.682)	*< 0.001*
CMR risk factor model				
MVO	1.880 (0.935–3.782)	0.08	–	–
LVEF < 52%	2.360 (1.209–4.608)	*0.01*	–	–
MAPSE < 9 mm	5.947 (2.595–13.630)	*< 0.001*	5.030 (2.108–12.003)	*< 0.001*

CI, confidence interval; MAPSE, mitral annular plane systolic excursion; CMR, cardiac magnetic resonance; MVO, microvascular obstruction; LVEF, left ventricular ejection fraction

**Fig. 2:**
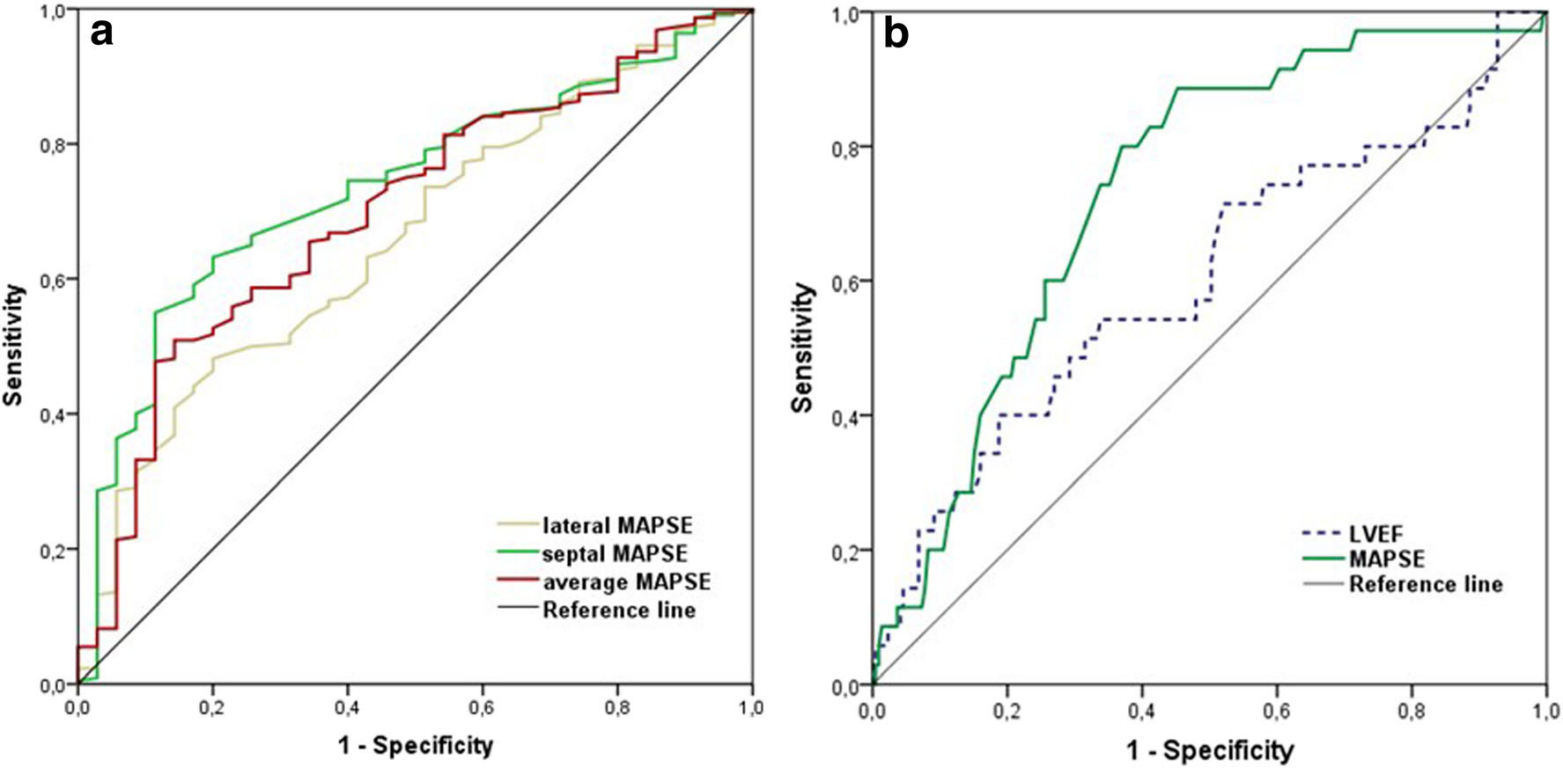
**a** ROC analysis of septal MAPSE (AUC 0.74 [95% CI 0.66–0.82]), average MAPSE (AUC 0.70 [95% CI 0.61–0.79]), and lateral MAPSE (AUC 0.66, [95% CI 0.57–0.75]) for the prediction of MACE. AUC indicates area under the curve; MAPSE, mitral annular plane systolic excursion; ROC, receiver operating characteristics. **b** ROC analysis of MAPSE (AUC 0.74 [95% CI 0.66–0.82]) and LVEF (AUC 0.60, [95% CI 0.50–0.78]) for the prediction of MACE. AUC, area under the curve; LVEF, left ventricular ejection fraction; MAPSE, mitral annular plane systolic excursion; ROC, receiver operating characteristics

Receiver operating characteristics (ROC) analysis revealed significantly higher (*p* = 0.03) AUC of septal MAPSE (0.74 [95% CI 0.66–0.82]) compared with average MAPSE (0.70 [95% CI 0.61–0.79]) as well as in comparison with lateral MAPSE (0.66, [95% CI 0.57–0.75], *p* = 0.01) for the prediction of MACE, see Fig. 2a.

The optimal cut-off value of septal MAPSE was 9 mm providing the highest sensitivity (80%) and specificity (63%) (Fig. 2b). This AUC of MAPSE was significantly higher (*p* = 0.03) than the AUC of LVEF (0.60 [95% CI 0.50–0.78]) for the prediction of MACE.

**Fig. 3:**
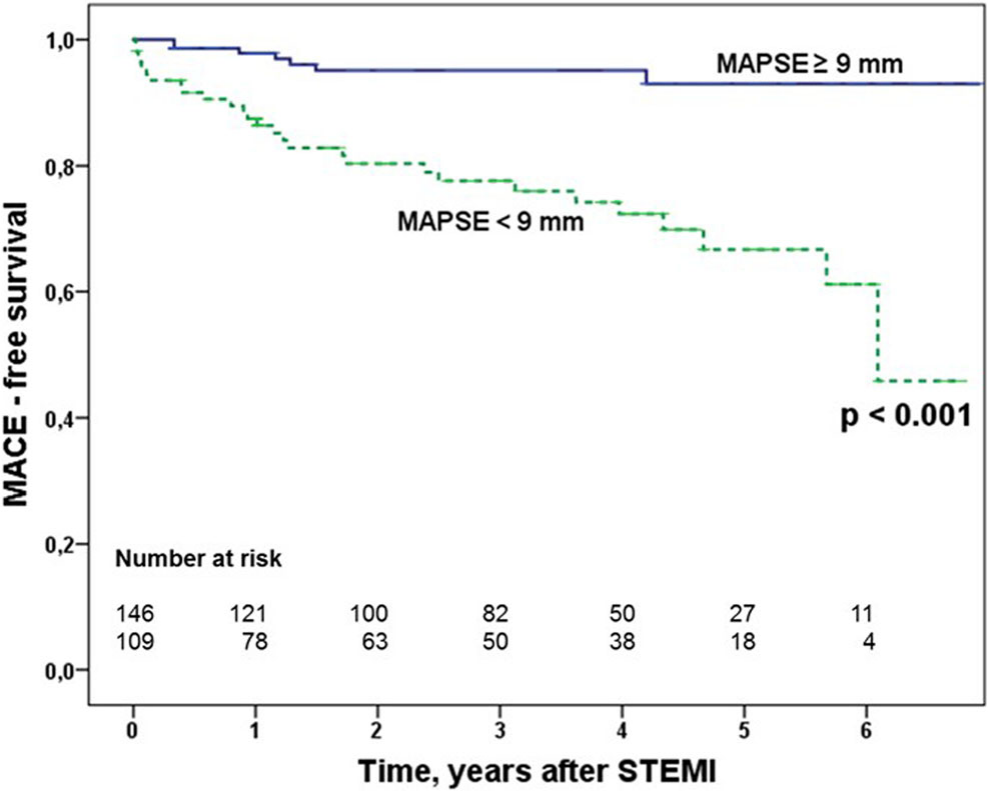
Kaplan–Meier curves for the occurrence of MACE stratified by ≥ and < 9 mm. MAPSE was calculated by ROC analysis. MACE indicates major adverse cardiovascular events; MAPSE, mitral annular plane systolic excursion; ROC, receiver operating characteristics

According to the Kaplan–Meier analysis, patients with MAPSE < 9 mm showed a significantly lower MACE-free survival (*p* = 0.001) than patients with MAPSE ≥ 9 mm (Fig. 3).

### Intra-observer and inter-observer variability for MAPSE measurements

Intra-observer analyses of septal MAPSE measurements showed an excellent agreement with an ICC of 0.94 (95%CI 0.75–0.98). The coefficient of variability was 13%. Similarly, inter-observer analyses showed excellent reproducibility of 0.89 (95%CI 0.50–0.98) and coefficient of variability 16%. The mean of the absolute difference between the two reader measurements was 0.57 mm. The mean of the absolute difference between the main reader measurements at different time points was 0.38 mm.

## Discussion

This study is the first to evaluate the prognostic value of CMR-derived MAPSE in a large STEMI cohort treated by pPCI. The main study findings can be summarized as follows:

1. Septal MAPSE determined by CMR early after reperfused STEMI is a powerful, independent predictor of MACE at long-term follow-up (3 years).
2. Patients with MAPSE ≥ 9 mm showed a significantly higher MACE-free survival than patients with MAPSE < 9 mm.
3. The predictive value of MAPSE was significantly higher than that of conventional LVEF.

Therefore, these results argue for the routine assessment of MAPSE in patients early after acute STEMI.

The longitudinal function of the LV is the main contributor to left ventricular pump function [6, 7]. Supposed longitudinal orientation of subendocardial myocardial fibers has long been discussed as a cause for impaired longitudinal function in patients with acute myocardial infarction [5, 18–20]. However, recent studies on myocardial microstructure assessed by CMR diffusion tensor imaging reported a spiral configuration of myocytes gradually shifting from a right-hand helix subendocardially to a left-hand helix subepicardially, working as a syncytium [21]. In myocardial infarction, myocardium adjacent to the scarred area shows a decrease in the right-hand helix of subendocardial fibers as well as a decreased range of helix angles across the whole myocardial wall in subacute myocardial infarction in a primate model [22]. Additionally, myocardial microstructures termed “sheetlets,” consisting of several myocytes, work together as mechanical units to change tilting angle over the cardiac cycle and are thought to be accountable for systolic myocardial thickening [21]. However, possible changes of sheetlet mechanics in myocardial infarction have not been studied yet. Therefore, underlying mechanisms of impaired longitudinal function in myocardial infarction are still to be discussed, as causality between myocardial fiber architecture and cardiac function has not been proven yet [23].

However, global longitudinal function remains the main contributor to LVEF in ischemic heart disease [5]. MAPSE as recorded by different echocardiographic methods, including M-mode, tissue Doppler, and strain imaging and speckle tracking assessed in both acute and chronic setting of myocardial infarction, was shown to predict adverse events [18–20, 24]. However, although easily available and cost-efficient, these techniques suffer from several disadvantages such as angle dependency issues for M-mode method or problems that are related to signal noise [25]. Strain imaging overcomes monodimensional focality by providing segmental and global information regarding longitudinal deformation but is highly dependent on imaging quality and operator experience [26]. Advanced CMR tools provide incremental prognostic stratification in patients with STEMI [27, 28] and easy-to-measure CMR-derived MAPSE has been linked to adverse outcome after STEMI. However, prognostic data of MAPSE in CMR either come from large all-comers cohorts without specific heart disease or from smaller groups of patients with myocardial infarction [10, 29, 30]. Our homogeneously treated a large patient group with first acute STEMI septal MAPSE with an optimal cut-off value of 9 mm provided the highest value for MACE prediction. Furthermore, septal MAPSE was shown to provide significantly higher predictive value after STEMI compared with lateral or average MAPSE. Other CMR studies revealed threshold values for lateral MAPSE of 9 mm [9] and 11 mm [10] respectively as cut-off points for risk stratification in mixed CMR populations. Lateral mitral annulus movement in healthy subjects is usually greater than septal movement (16 ± 3 mm compared with 13 ± 3 mm) [31]. Therefore, a larger cut-off value for lateral MAPSE is in accordance with anatomical properties. Romano et al used death as a primary endpoint, which might serve as an explanation why their reported cut-off for lateral MAPSE (9 mm) is equal as we found for septal MAPSE. However, Pahlm et al reported a decrease in global and regional MAPSE in infarcted as well as remote myocardium, supporting our thesis that septal MAPSE provides prognostic information regardless of infarct localization [32]. Based on the significantly higher predictive value of septal MAPSE compared with lateral and average MAPSE in our population as well as on the independency of MAPSE decrease from infarct location, we consider the exclusive measurement of septal MAPSE to be reasonable.

Numerous studies have demonstrated LVEF as a marker of global systolic myocardial function and a powerful predictor of morbidity and mortality in patients with acute reperfused myocardial infarction [33, 34]. Recently presented CMR indices and scores integrating several structural and/or morphological variables were suggested to provide incremental prognostic validity in STEMI patients [27, 35]. However, septal MAPSE is a unique, simple measurable and effective marker in standard CMR and our study showed that it offers prognostic information that adds beyond traditional clinical and imaging cardiac risk factors. Moreover, the high reproducibility of MAPSE measurements shown by Romano et al was confirmed in our study by a very low intra- and inter-observer variability [29]. The superior prognostic performance of MAPSE compared with that of LVEF in STEMI patients may be explained by the suspicion that longitudinally running myocardial fibers are located subendocardially and are most affected by ischemia. However, LVEF primarily tracks radial function of the myocardium. Anyhow, recent studies suggest more complex organization of cardiomyocyte microstructure and dynamics [21]. In accordance with the findings previously reported by van Kranenburg et al, infarct size in our study was not significantly associated with MACE occurrence [36]. To what extent large but only subendocardial infarcts are less favorable for long-axis function and prognosis than smaller but transmural infarcts remains to be investigated.

Despite rapid reperfusion of epicardial coronary circulation by pPCI in STEMI, severe microvascular dysfunction, related to initial ischemia and/or to reperfusion injury, may persist [37]. MVO is a significant and independent short- and long-term prognosticator for morbidity and mortality after STEMI [38, 39]. In our current study, patients with MVO showed a trend towards higher incidence of MACE but did not reach significance (*p* = 0.07). One explanation could be that our definition of adverse outcome included stroke (23% of total MACE). Previous studies only included death and cardiac events (re-infarction, hospitalization for heart failure) [36, 38].

Specialized CMR techniques such as strain-encoded MR (SENC) [39] and feature-tracking software promise risk stratification in patients with various cardiac diseases [28, 40–45]. Several studies recently assessed the relationship of LV strain, infarct characteristics such as edema or hemorrhage, and their association with prognosis in patients with acute myocardial infarction; while Eitel et al [45] highlighted CMR feature tracking to have incremental prognostic value above LVEF and infarct size, others [41] showed tissue tracking to not substantially improve risk reclassification beyond LVEF, infarct size, and MVO. However, this technique requires the use of specialized software and consequently has not achieved widespread clinical use [46]. Moreover, strain imaging is difficult to measure in the acute setting, and there is a lack of evidence for its benefit. Good image quality is an essential prerequisite for strain analysis as well as for LVEF measurement while in case of poor imaging quality, MAPSE could still be a suitable choice for assessing longitudinal function.

Septal MAPSE is simple to measure on 4-chamber routine cine images, available from any vendor without any specific software and with a good inter- and intra-observer variability.

## Limitations

Due to our inclusion criteria, the results of this study only apply to a selected patient group and must not be generalized for patients with recurrent myocardial infarction or other cardiac pathologies that entail left ventricular remodeling. However, with an overall incidence rate of STEMI of 43 to 144 per 100,000 in Europe [47], specific data for this particular group should be available to support clinical decision-making.

We did not measure atrial volumes or left atrial functional parameters for the prediction of outcome after STEMI as it was suggested before [48]. Future studies may address the left atrium parameters. We only measured absolute values of septal MAPSE and did not take into account factors like patient size, ventricular diameters, sex, or age that might influence absolute mitral plane motion [49, 50]. Upcoming technologies such as SENC imaging and new feature-tracking software have not been evaluated. However, their clinical relevance is still controversial [41].

## Conclusion

We provide a parameter that is simple to measure and easy to implement in clinical routine without the need for specialized devices or software. Reduced septal MAPSE <9 mm—assessed by cine CMR—is an independent long-term predictor of MACE in patients after first-time STEMI undergoing pPCI. It provides superior prognostic value compared with LVEF.

## Abbreviations

AUC: Area under the curve
CMR: Cardiac magnetic resonance
CoV: Coefficient of variation
HR: Hazard ratio
ICC: Intraclass correlation coefficients
IQR: Interquartile range
IS: Infarct size
LV: Left ventricular
LVEF: Left ventricular ejection fraction
MACE: Major adverse cardiac events
MAPSE: Mitral annular plane systolic excursion
MVO: Microvascular obstruction
pPCI: Primary percutaneous coronary intervention
SD: Standard deviation
SENC: Strain-encoded magnetic resonance
STEMI: ST-elevation myocardial infarction
